# Association between Air Pollution and Hemoptysis

**DOI:** 10.1155/2016/9242185

**Published:** 2016-03-07

**Authors:** Ignasi Garcia-Olive, Joaquim Radua, Jose Antonio Fiz, Jose Sanz-Santos, Juan Ruiz-Manzano

**Affiliations:** ^1^Respiratory Department, Hospital Universitari Germans Trias i Pujol, Carretera del Canyet, s/n, Badalona, 08916 Barcelona, Spain; ^2^Ciber de Enfermedades Respiratorias (CIBERES), Carretera Soller km 12, Bunyola, 07110 Mallorca, Spain; ^3^Fundació Institut d'Investigació en Ciències de la Salut Germans Trias i Pujol, Carretera del Canyet, s/n, Badalona, 08916 Barcelona, Spain; ^4^Department of Statistics, FIDMAG Research Unit, Sant Boi de Llobregat, 08830 Barcelona, Spain; ^5^Ciber de Salud Mental (CIBERSAM), 28035 Madrid, Spain; ^6^Departament de Medicina, Universitat Autònoma de Barcelona, Bellaterra, 08193 Barcelona, Spain

## Abstract

*Background*. The relationship between air pollution and exacerbation of respiratory diseases is well established. Nevertheless, its association with hemoptysis has been poorly investigated. This paper describes the relationship of air pollutants with severe hemoptysis.* Methods*. All consecutive subjects with severe hemoptysis during a 5-year period were included. The relationship between the contamination measurements and the frequency of embolizations was analyzed using Poisson regressions. In these regressions, the dependent variable was the monthly number of embolizations in a given month and the independent variable was either the concentration of an air contaminant during the same month, the concentration of the air contaminant during the previous month, or the difference between the two.* Results*. A higher total number of embolizations per month were observed over the months with increases in the concentration of NO. The number of embolizations was 2.0 in the 33 months with no increases in the concentration of NO, 2.1 in the 12 months with small increases, 2.2 in the 5 months with moderate increases, 2.5 in the 4 months with large increases, and 4.0 in the 5 months with very large increases.* Conclusion*. There is association between hemoptysis and increases in the concentration of atmospheric NO in Badalona (Spain).

## 1. Introduction

Hemoptysis is a potentially life-threatening situation that requires prompt intervention. Several conditions such as tuberculosis, cancer, or bronchiectasis may lead to clinically significant hemoptysis. Some authors have suggested that there is some seasonal periodicity of hemoptysis [1–3] or have described its association with respiratory tract infections [4–7], or with climatic parameters [7].

The relationship between ambient air pollution and exacerbation of respiratory diseases [8–11] or lung function decline [12, 13] is well established. Nevertheless, the association of air pollution with life-threatening hemoptysis has been poorly investigated.

This paper describes the relationship of air pollutants with severe hemoptysis that required bronchial artery embolization (BAE).

## 2. Methods

### 2.1. Institution

This was an observational retrospective study of subjects presenting with life-threatening hemoptysis that underwent BAE at Hospital Universitari Germans Trias i Pujol (HGTiP), a 600-bed tertiary referral hospital in Badalona (Catalonia, Spain), a referral hospital for over 700,000 people. In 2009 there were 27,000 hospital admissions and 110,000 admissions in the emergency room. The closest reference hospital that can perform embolizations is located 10 km away.

### 2.2. Case Definition

All consecutive subjects with at least one episode of life-threatening hemoptysis that required BAE during a 5-year (January 2007–December 2011) period were included. Indication for BAE was life-threatening hemoptysis, which was defined as either bleeding of 200 mL during 24 hours or 100 mL daily during at least three days or a minor hemoptysis with hemodynamic instability. Before BAE all patients had a contrast enhanced CT of the chest. Recurring hemoptysis requiring BAE in a subject with previous embolization was included as a new hemoptysis event, unless it occurred within one month from the prior event (this was considered as an early recurrent bleeding) [14]. We obtained the number of BAE for every month of the year during the study period.

Those subjects residing farther than 15 km away from our institution were not included in the analysis to ensure that they lived close to the atmospheric pollution control sampling station.

### 2.3. Air Pollution Data Collection

Catalonia has a network of atmospheric pollution control sampling stations operated by the Department of Environment of the Catalan Autonomous Government. Air pollution data were obtained from a single station located in Badalona, 5 km away from our institution, from Jan 2007 to Dec 2011 (http://www20.gencat.cat/portal/site/mediambient/ accessed July 13, 2014). This station is the closest atmospheric pollution sampling station to the hospital. Monthly mean values were obtained for sulfur dioxide (SO_2_), nitric oxide (NO), nitrogen dioxide (NO_2_), ozone (O_3_), carbon monoxide (CO), and particulate matter with a diameter of <10 *μ*m (PM_10_). Information on other pollutants such as PM_2.5_ was not available.

### 2.4. Influenza Activity

The Internet search engine Google developed Google Flu Trends (GFT) in 2008, to estimate national and regional influenza incidence. GFT is highly correlated with historical influenza-like illness (ILI) conventional surveillance data and can detect regional outbreaks of influenza 7–10 days earlier than the existing US CDC surveillance system [15].

For the purpose of this study, the weekly GFT estimates of influenza incidence in Catalonia, a northeastern region of Spain with an area of 32,114 km^2^, were recorded (http://www.google.org/flutrends accessed January 10, 2013), and monthly estimates were calculated. Although our institution is a referral hospital for a smaller area, there was no other source that could be used to validate the influenza activity in the area covered by our institution; therefore we assumed that the GFT estimates for Catalonia correlated with influenza activity in our area.

### 2.5. Climatic Data Collection

Monthly mean temperature was obtained from the Catalan meteorological agency (http://www.meteo.cat/ accessed December 13, 2012) for a single weather station located in Badalona, 5 km away from our institution from Jan 2007 to Dec 2011.

### 2.6. Statistical Analysis

The relationship between the contamination measurements and the frequency of embolizations was analyzed using simple Poisson regressions. In these regressions, the dependent variable was the monthly number of embolizations in a given month and the independent variable was either (a) the concentration of an air contaminant during the same month (*x*_*t*_), (b) the concentration of the air contaminant during the previous month (*x*_*t*−1_), or (c) the difference between the two (*x*_*t*_ − *x*_*t*−1_, i.e., the increase in the concentration). Potential collinearity between any pair of pollutants as well as those between the monthly concentrations of a pollutant and the difference of concentration between the same and the previous month was checked using the variance inflating factor (VIF). Variables with VIFs of 5 or above would be considered to be affected by collinearity. The number of days of each month was included as an offset to model the longer exposure during 31-day months compared to that during 28-to-30-day months. Bonferroni correction for multiple comparisons accounted for the fact that this study assessed the effect of six different air contaminants.

Prior to conducting these regressions, the monthly number of embolizations was checked to follow the theoretically expected Poisson distribution by visually inspecting the histogram and conducting a *χ* ^2^ goodness of fit test. Similarly, it was checked to not be overdispersed or zero-inflated by ensuring that the variance was not substantially larger than the mean (indicating overdispersion) and that there was no excess of zeros (indicating zero-inflation). The potential effects of seasonality were assessed by fitting Poisson regressions by the sine and cosine functions of the month [16], so that a gradual shift from month to month was modeled: sin⁡[(month − phase_1_) × 2*π*/12] and cos⁡[(month − phase_2_) × 2*π*/12].

The potential effects of both monthly temperature and monthly influenza virus activity were assessed by fitting simple Poisson regressions with these factors as independent variables. Statistically significant potential confounds and interactions could be added to the Poisson regressions following a forward stepwise strategy. Calculations were conducted in R [17].

### 2.7. Ethical Considerations

The research protocol was approved by the regional ethics committee (Ethics Committee for Clinical Research of the Hospital Germans Trias i Pujol).

## 3. Results

One hundred twenty-five subjects who underwent 133 embolizations were included in the analysis (Table 1), with 27 of them (21%) having been diagnosed as having idiopathic hemoptysis, that is, when no underlying etiology (such as pulmonary diseases, pulmonary embolism, or cardiac disease) could be discovered.

Distribution of monthly embolization data did not deviate from the theoretically expected Poisson distribution (*χ* ^2^ = 1.97, *p* = 0.922) and no overdispersion or zero-inflation was observed (variance/mean = 0.95; observed versus expected number of zeros: 5 versus 6.3), thus indicating the adequacy of Poisson regressions. Seasonality, temperature, and influenza virus activity did not show any statistically significant effect (all *p* > 0.065) and no pairwise collinearity was detected among pollutant concentrations.

A higher total number of embolizations per month were observed over time during the months with increases in the concentration of NO with respect to the previous month one (*p* = 0.006, corrected *p* = 0.034, Table 2). To provide a straightforward idea of the strength of the relationship we calculated the number of embolizations per month to be 2.0 in the 33 months with no increases in the concentration of NO (*x*_*t*_ − *x*_*t*−1_ ≤ 0), 2.1 in the 12 months with small increases (0 < *x*_*t*_ − *x*_*t*−1_ ≤ 5 *μ*g/m^3^), 2.2 in the 5 months with moderate increases (5 < *x*_*t*_ − *x*_*t*−1_ ≤ 10), 2.5 in the 4 months with large increases (10 < *x*_*t*_ − *x*_*t*−1_ ≤ 15 *μ*g/m^3^), and 4.0 in the 5 months with very large increases (*x*_*t*_ − *x*_*t*−1_ > 15 *μ*g/m^3^). In order to provide a more comprehensive study of these effects, we checked for potential interactions with seasonality, temperature, and influenza activity. A potential negative interaction was found with monthly temperature (*p* = 0.012): the Poisson regression exponentiated coefficient per 15 *μ*g NO/m^3^ increase was estimated to be around 2 (i.e., 100% increase in the number of embolizations) in the coldest months (mean temperature ≤10°C) and around 1 (i.e., no relationship) in warmer months (mean temperature ≥20°C).

A similar effect was found when analyzing the concentration of NO_2_, although the relationship was no longer significant after adjusting for multiple comparisons (*p* = 0.014, corrected *p* = 0.085).

## 4. Discussion

This study provides evidence of an association between severe hemoptysis requiring BAE and increases in the concentration of atmospheric NO with respect to the previous month in Badalona (Spain). A similar association was found when analyzing the concentration of NO_2_, though the relationship was not statistically significant after adjusting for multiple comparisons. We found no relationship between the other pollutants analyzed (SO_2_, O_3_, CO, and PM_10_) and the incidence of life-threatening hemoptysis.

Air pollution is a known risk factor for poor outcomes and exacerbations in both respiratory [8–12, 18] and nonrespiratory diseases [19–23]. Recently, Johannson et al. [9] have described that increased ozone and nitrogen dioxide exposure over the preceding six weeks was associated with an increased risk of acute exacerbation of idiopathic pulmonary fibrosis. Another group suggested that traffic-related air pollution was likely to increase the risk of death in patients with noncystic fibrosis bronchiectasis [10]. Nevertheless, they did not measure air pollutants but instead used the distance to a road and its traffic as indicators of exposure. Yorifuji et al. [11] evaluated the association between hourly changes in air pollution and the risk of respiratory disease in the elderly. They found that suspended particulate matter (SPM) exposure 24 to 72 hours prior to the onset and ozone exposure 48 to 96 hours prior to the onset were associated with increased risk of respiratory disease. SO_2_ exposure 0 to 24 hours prior to the onset was associated with increased risk of pneumonia and influenza. Additionally, Lepeule et al. [12] evaluated the effect of long-term exposure to black carbon on levels and rates of decline in lung function in the elderly. Their results suggested the existence of adverse effects of long-term exposure to this pollutant. Nevertheless, to the best of our knowledge, no study has to date evaluated how air pollution correlates with hemoptysis.

The mechanism by which air pollutants damage the lungs and increase exacerbations is not completely clear. A plausible biomedical explanation would be the activation of inflammatory pathways in the small airways in response to particulate and gaseous pollutant exposure, resulting in the recruitment of inflammatory cells and the generation of inflammatory mediators [19–23]. Particles, NO_2_, and O_3_ are oxidants that can trigger intracellular oxidative stress [13, 24, 25], causing clinical effects in those individuals who are more vulnerable due to existing chronic or acute disease. Several studies have shown wide interindividual variability in responses to air pollutants. Although genetic factors surely influence this variability, some other factors must be taken into consideration, such as the distribution of ventilation in the different lung compartments and in the case of particle differences in regional depositions within the lungs [8].

Nitrogen oxides (NO_*x*_), a mixture of NO and NO_2_, are produced from natural sources, motor vehicles, and other fuel combustion processes. NO is not known to significantly affect human health, but there is a concern regarding NO emission and levels [26]. In ambient conditions, NO is rapidly transformed into NO_2_ by atmospheric oxidants such as ozone.

Many studies have documented associations between day-to-day variations in NO_2_ concentration and variations in mortality, hospital admissions, and respiratory symptoms. The associations between NO_2_ and short-term health effects in many studies remain after adjustment for other pollutants (mainly PM_10_ and sometimes PM_2.5_ or black smoke) [27]. Accidental exposure to NO_2_ in indoor ice arenas has also been described. The symptoms included cough, shortness of breath, hemoptysis, and chest pain or tightness [28, 29].

Identification of significant triggers of hemoptysis, such as low temperature or air pollution, could be useful for physicians to improve prevention measures and educational strategies in those patients with those diseases that are more likely to produce hemoptysis, or those with a higher risk of recurrence. Air pollution is a potentially modifiable risk factor either by exposure avoidance or through environmental policy.

This study has several limitations. First of all, we used a therapeutic technique as an indirect indicator of hemoptysis. Thus, not all patients with hemoptysis are included in this study, which would have been desirable. Unfortunately, we could not provide the data regarding all the patients with hemoptysis during the study period who had not undergone BAE. Those who died due to massive hemoptysis prior to BAE or those with mild or moderate hemoptysis that did not require BAE were not included in the study. Secondly, we used outdoor air pollution exposure measured at a distance of 5 km from our institution to estimate individual exposure levels, which may not reflect actual exposures. Third, data on the medications used by patients was not available and therefore could not be included in the analysis. This would have been of interest, especially in the case of anticoagulants. And lastly, daily values of air pollutants were not available, so it was not possible to analyze the effect of peak levels of these pollutants on severe hemoptysis.

The main strength of this report is that all patients included in the study had confirmed severe hemoptysis, and other conditions that could be considered as hemoptysis (such as hematemesis) have been excluded.

## 5. Conclusion

In summary, we found that increases in concentration of air NO were associated with the risk of severe hemoptysis requiring BAE, and we speculate that air pollution represents one of a series of factors that serve as triggers for this clinical event. Confirmatory studies on the association of air pollution with hemoptysis shown in the present study are desirable, utilizing larger data sets.

## Figures and Tables

**Table 1:** Characteristics of the 125 subjects included in the study.

Age (years)	
Median, mean (SD)	60.6, 58.2 (17)
Sex (*n* (%))	
Male	92 (74)
Female	33 (26)
Pathological condition (*n* (%))	
Bronchiectasis	41 (33)
Cancer	25 (20)
COPD	22 (18)
Active tuberculosis	10 (8)
Idiopathic	27 (21)
Embolizations per patient (*n* (%))	
1	115 (92)
2	8 (6)
≥3	2 (2)

**Table 2:** Poisson regression exponentiated coefficients of the relationship between concentration of air contaminants and monthly number of embolizations.

Relationship between number of embolizations in a given month and …	… concentration of the contaminant in the same month (*x*_*t*_)	… concentration of the contaminant in the previous month (*x*_*t*−1_)	… increase (from the previous month) in the concentration of the contaminant (*x*_*t*_ − *x*_*t*−1_)
Contaminants			
SO_2_ (per 1 *μ*g/m^3^)	1.010 (*p* = 0.918)	1.070 (*p* = 0.521)	0.961 (*p* = 0.636)
NO (per 15 *μ*g/m^3^)	1.169 (*p* = 0.132)	0.958 (*p* = 0.679)	1.445 (*p* = 0.006)^*∗∗*^
NO_2_ (per 10 *μ*g/m^3^)	1.135 (*p* = 0.177)	0.945 (*p* = 0.521)	1.292 (*p* = 0.014)^*∗*^
O_3_ (per 20 *μ*g/m^3^)	0.868 (*p* = 0.154)	0.900 (*p* = 0.301)	0.839 (*p* = 0.302)
CO (per 0.1 *μ*g/m^3^)	1.073 (*p* = 0.490)	0.923 (*p* = 0.511)	1.253 (*p* = 0.094)
PM_10_ (per 10 *μ*g/m^3^)	1.029 (*p* = 0.770)	0.991 (*p* = 0.926)	1.097 (*p* = 0.432)
Potential confounds			
Temperature (per 10°C)	0.987 (0.933)	1.118 (0.511)	0.596 (0.101)
Influenza (per 500 searches)	0.808 (0.182)	0.857 (0.339)	0.993 (0.959)

Each cell shows the Poisson regression exponentiated coefficient and the *p* value of the relationship. The exponentiated coefficient shows the amount by which the expected number of embolizations is multiplied per each unit increase in the contaminant (e.g., for the right-most NO cell, 1.169, the expected number of embolizations in a given month is 16.9% higher if the concentration of NO is 15 *μ*g/m^3^ higher). The measurement units were semiconventionally chosen (round numbers with similar standard deviations ~ 0.5–1), but statistics were conducted considering the variables as continuous for which  *p* values do not depend on these measure units.

^*∗∗*^Statistically significant after correction for multiple comparisons.

^*∗*^Only statistically significant before correction for multiple comparisons.
